# A Curious Researcher’s Guide on Successfully Publishing Scientific Manuscripts

**DOI:** 10.7759/cureus.2683

**Published:** 2018-05-24

**Authors:** Xiao Chi Zhang, Alexander H Tran, Dimitrios Papanagnou

**Affiliations:** 1 Department of Emergency Medicine, Thomas Jefferson University, Philadelphia, USA; 2 Medical School, Alpert Medical School of Brown University , Providence, USA

**Keywords:** medical education, research, mentoring, publication

## Abstract

Publishing a manuscript in an academic journal represents more than just ‘promotional currency.’ It provides the opportunity to provoke debate, share your experiences, and challenge the status quo on provider practices. Writing a manuscript relies on collaboration and shared responsibility from a research team, which can often challenge mentors as they supervise and guide its development. While there are numerous online resources and peer-reviewed journal articles on ‘How to write a scientific article,’ we aim to tackle an even larger and overarching theme that transcends specific journal categories, writing styles, and citation formatting. In order to guide new researchers in navigating the expansive ocean of scientific publications, we collected, reviewed, and revised 11 tips based on multiple focus-group discussions with junior and senior researchers to determine common barriers for publishing manuscript. The goal is to help mentors and mentees navigate the research and publication processes. The tips include: 1) recognizing leadership styles; 2) initiating the groundwork; 3) establishing backup plans; 4) making a deadline; 5) courting the editor; 6) determining authorship; 7) finding personal incentives; 8) writing what you know; 9) sharing with caution; 10) following directions; and 11) Consider open-access journals.

## Editorial

‘Wanna write that up?’

As you smile beamingly toward your research mentee reflecting on the outcomes of your recent study, you begin to notice large beads of sweat developing on his forehead. There are few phrases in emergency medicine (EM) that can induce as much intimidation and even visceral pain as these four simple words strung together.

But why does this have to be the case? Publishing a manuscript in an academic journal represents more than just ‘promotional currency’ or another line on your curriculum vitae. It provides the opportunity to provoke debate, share your experiences, and challenge the status quo on provider practices. Writing a manuscript relies on collaboration and shared responsibility from a research team, which can often challenge mentors as they supervise and guide its development.

As a junior faculty in EM, I have had the experience with researchers from various levels of training, from medical students to residents and fellows, and I have encountered, both personally and vicariously through my research colleagues, the significant amount of fear and resistance to writing scientific manuscripts. While there are numerous online resources and peer-reviewed journal articles on ‘How to write a scientific article,’ we aim to tackle an even larger and overarching theme that transcends specific journal categories, writing styles, and citation formatting.

**Figure 1:**
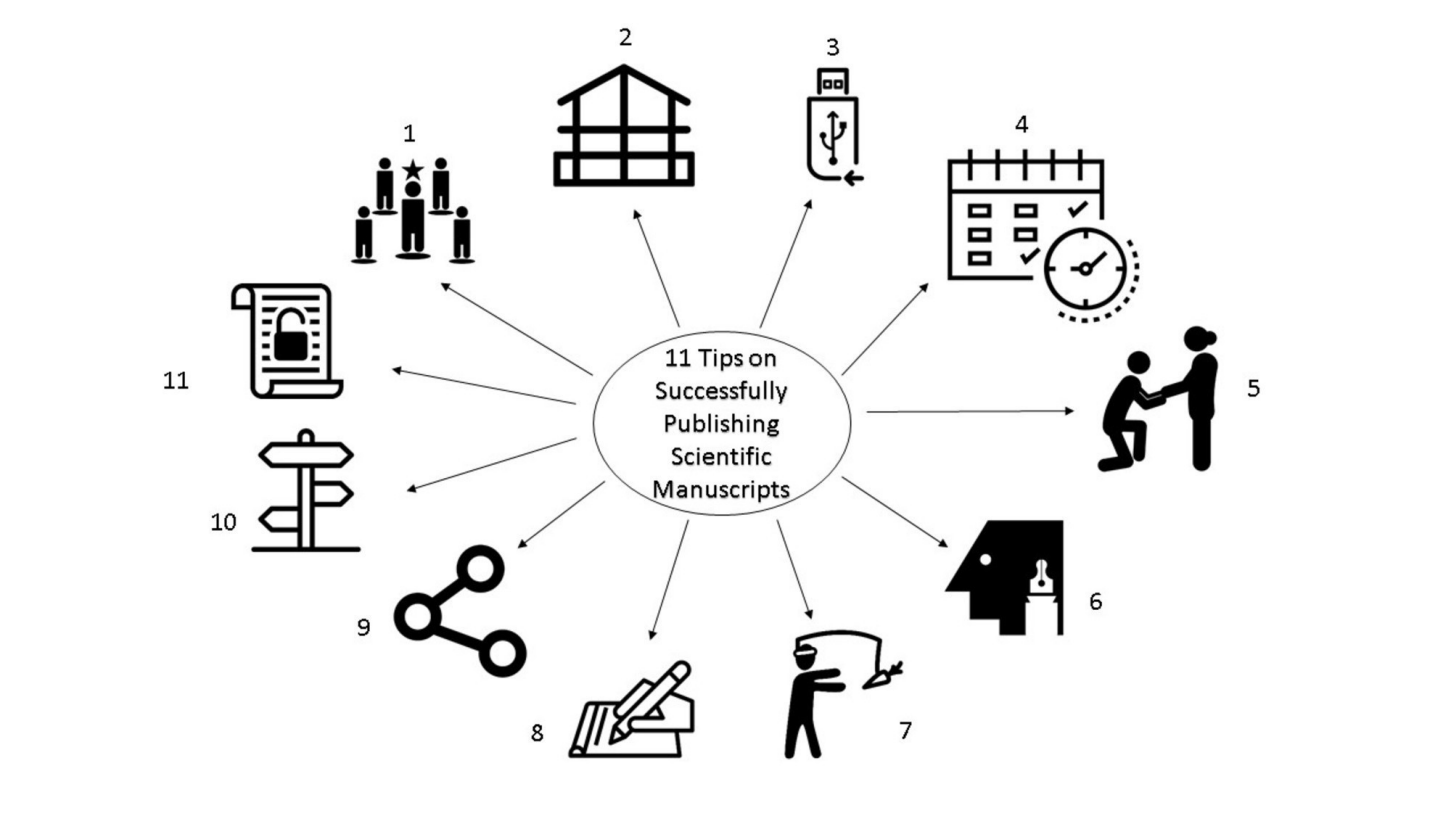
Eleven Tips to Successfully Publishing Scientific Manuscripts All icons are copyright-free and available from https://thenounproject.com/.

In order to guide new researchers in navigating the expansive ocean of scientific publications, we collected, reviewed, and revised 11 tips based on multiple focus-group discussions with junior and senior researchers to determine common barriers for publishing manuscripts (Figure 1). The goal is to help mentors and mentees navigate the research and publication processes. The conceptual framework for these steps follows the basic guidelines behind the popular SMART [1] acronym for goal setting (Specific, Measurable, Achievable, Relevant, and Time-bound), and the path-goal leadership theory [2].

Eleven tips on successfully publishing scientific manuscripts

For Mentors

1. Recognizing your leadership styles: Each research team is often composed of a senior mentor (aka. Project Lead), primary author, and co-authors. As the mentor, it is imperative to recognize your leadership characteristics to optimize your interactions with your co-authors. Common leadership styles include contingency, exchange, path-goal, laissez-faire, situational, transformational, transactional, and servant [2-3]. You can check to see which style fits you by exploring cost-free commercially available tests, such as the Mark Murphy Leadership IQ Test [4].

2. Initiate the groundwork: One of the most crucial elements as a research mentor is to provide the necessary groundwork and articulate specific and tangible expectations for all responsible stakeholders (i.e., the authors) during the write-up. Consider the following questions your mentees may ask, and prepare for constructive feedback and guidance: 1) How does this research impact the scientific community? Is this an original study? What is your elevator pitch?; 2) Are there any particular journal preferences for publication?; 3) Which category (i.e., original research, technical report, brief report) should this manuscript be written in?; 4) How often should the team meet to review progress?; 5) Are there any institutional research personnel (i.e., statistician, librarian) who can assist with the data analysis?; 6) Is there any supporting literature regarding the research topic?; 7) What is the backup plan if the manuscript gets rejected?

3. Establish early backup plans: Specifying particular backup plans, such as revision or resubmission to another journal, can alleviate some of the stress from your fellow authors by ensuring multiple paths for publication. Editors receive countless submissions on a regular basis; so, in the event, your submission gets rejected, your co-authors know what the next steps will be for the manuscript (i.e., revision or resubmission to another journal). Remind your team to not give up and make it clear that this is always an expected outcome. For example, if Journal A doesn’t accept, then reformat and resubmit to Journal B, followed by Journal C, and finally Journal D.

4. Make a deadline and stick to it!: Having a reasonable timeline allows all members of the team to contribute their weight and justify their authorship. The key is to have a group consensus with an agreed-upon deadline and send it via a traceable platform (i.e., email) so it can be readily referenced. Common deadline milestones include 1) completion of the literature search; 2) completion of the data analysis; 3) draft of the abstract; 4) manuscript draft; 5) abstract revision and submission in journal format; and quite likely 6) manuscript revision and resubmission. 'Tomorrow' may be variable, but deadlines are always constant.

For Co-authors

5. Courting the editor: The next step should be to pitch your manuscript submission to the editor-in-chief of the journal of interest. A well-crafted ‘would you be interested in . . . ' letter may peak editors’ interests and potentially save you hours of formatting your manuscript if the editors were not interested in your work.

6. Determine authorship order early: Given that first or last authorship on a publication is often considered to be of high value towards academic promotion, physicians interested in pursuing an academic career should be mindful of both authorship considerations and authorship etiquette. This should not minimize the efforts of other authors, as being an author on any paper will be beneficial to one’s academic career. The primary author is responsible for collating the authors’ inputs into a scientific deliverable [5]. The senior author, who is often the research mentor, is responsible for coordinating the writers and providing appropriate incentives for timely completion, as well as editing.

7. Find your personal incentive: To ensure a teamwork and a smooth process to packaging your scholarship, it is imperative to motivate your team with the proverbial ‘incentive carrot.’ Discovering your respective incentives early during the write-up can ensure prompt and timely submissions. Here are some common publication incentives: 1) Departmental promotion (mentor); 2) Residency requirement (i.e., scholarly project); 3) Departmental funding to attend a conference for abstract acceptance(s) and/or presentations(s); 4) Curriculum vitae builder.

8. Start writing what you know: Sometimes the best way to build momentum and overcome writer’s block is to start writing what you already know (i.e., the introduction or methodology). A common tool to alleviate the stress associated with starting to write your manuscript is to consider leveraging sections from the Institutional Review Board (IRB) application that was submitted at your sponsoring institution. Always, save the abstract for last; it’s often the most challenging part of any manuscript and will require that you have reflected on the other sections of your paper.

9. Sharing is caring – but use with caution: Shared documents (i.e., Google Documents) allow authors to contribute to the submission in real time; however, it can also result in procrastination. Additional challenges to shared documentation include citation placements, which are compiled in piecemeal fashion in a free-form sharing model. As a result, cloud-sharing and editing should be considered only after the initial rough draft is compiled in order to allow for a revision. There are numerous institutionally-provided, free citation software programs, such as Mendeley, which can streamline the integration of your citations.

10. Follow directions!: You must ensure that your paper fulfilled all of the journal’s requirements, including, but not limited, to word count, margin size, font, citation style, and/or image limits. A submission that is riddled with errors, formatting faux-pas, or poorly reference citations may be permanently rejected.

11. Consider open-access journals: Once you get published, you are going to want readers to notice your work, share your ideas, and maximize your regional impact. Unfortunately, however, unless readers have access to the journal you are published in, they are not going to have access to your submission. Open-access journals are readily available online to all readers and do not require a subscription fee to access articles. With improved access to content, individuals in your academic circle (including the general public) can learn more about your work and even cite you in subsequent scholarly publications. If you want to get noticed, and if you do not mind having your full article appear in Google Scholar, then consider submitting your manuscript to an open-access journal.

Ready, set, publish!

Now that you’ve survived reading this research guide, dust-off your work and start writing it up by first building your team and creating a plan to get it completed in a timely fashion. Most importantly, do not forget to motivate your team members and have them take pride in their scientific achievements as they declare their findings to the rest of the academic world.
